# Supplementary material for: Cross-Sectional and Longitudinal Associations between Non-School Time Physical Activity, Sedentary Time, and Adiposity among Boys and Girls: An Isotemporal Substitution Approach
Source: Int J Environ Res Public Health. 2021 Apr 27;18(9):4671. doi: 10.3390/ijerph18094671 (PMC8125309; doi:10.3390/ijerph18094671)
Supplement: Supplementary file 1 [file ijerph-18-04671-s001.zip › ijerph-1179935-supplementary.pdf]

International Journal of
Environmental Research and Public Health

MDPI

Supplementary Tables S1a–d Cross-sectional and longitudinal associations between 15-minute reallocations of non-school time LPA, MVPA, and ST and BMI, WHR, and BF% in boys and girls separately

Table S1a. Cross-sectional replacement effects (β [95% CI]) at follow-up of substituting 15 minutes of non-school time LPA, MVPA, and ST on BMI, WHR, and BF% in boys (n = 65).

		BMI [a]		
	With 30 min/day of:			
Replacing 30 min/day of:	LPA	MVPA	ST	Total wear-time
LPA	--	**-1.40**<br>**[-3.02, 0.21]** #	**-0.49**<br>**[-1.08, 0.09]** #	**0.39**<br>**[-0.09, 0.86]** #
MVPA	**0.07**<br>**[-0.01, 0.14]** #	--	0.04<br>[-0.01, 0.10]	-0.05<br>[-0.10, 0.01]
ST	**0.49**<br>**[-0.09, 1.08]** #	-0.04<br>[-0.10, 0.01]	--	-0.01<br>[-0.02, 0.05]
		WHR		
	With 30 min/day of:			
Replacing 30 min/day of:	LPA	MVPA	ST	Total wear-time
LPA	--	**-0.02**<br>**[-0.04, 0.004]** #	**-0.005**<br>**[-0.01, 0.003]** #	0.004<br>[-0.002, 0.01]
MVPA	**0.02**<br>**[-0.004, 0.04]** #	--	0.01<br>[-0.004, 0.03]	-0.02<br>[-0.03, 0.003]
ST	**0.005**<br>**[-0.003, 0.01]** #	-0.01<br>[-0.03, 0.004]	--	-0.001<br>[-0.004, 0.002]
		BF% [b]		
	With 30 min/day of:			
Replacing 30 min/day of:	LPA	MVPA	ST	Total wear-time
LPA	--	**-4.13**<br>**[-7.71, -0.55]** *	**-1.12**<br>**[-2.43, 0.19]** #	0.86<br>[-0.23, 1.94]
MVPA	**4.13**<br>**[0.55, 7.71]** *	--	**3.01**<br>**[0.24, 5.78]** *	**-3.27**<br>**[-6.15, 0.39]** *
ST	**1.12**<br>**[-0.19, 2.43]** #	**-3.01**<br>**[-5.78, -0.24]** *	--	-0.26<br>[-0.78, 0.26]

Bold estimates represent significant or marginally significant associations. Abbreviations: min: minutes; CI: confidence interval; LPA: light physical activity; MVPA: moderate-to-vigorous physical activity; ST: sedentary time; BMI: body mass index; WHR: waist-to-height ratio; BF%: body fat percent. All models were adjusted for child age, ethnicity, mother's education, and accelerometer wear-time. [a] Log BMI was used to satisfy model assumptions. Estimates are presented in the log transformation of BMI [b]; n = 59. * $p < 0.05$; # $p < 0.10$.

Table S1b. Cross-sectional replacement effects (β [95% CI]) at follow-up of substituting 15 minutes of non-school time LPA, MVPA, and ST on BMI, WHR, and BF% in girls (n = 77).

		BMI [a]		
	With 30 min/day of:			
Replacing 30 min/day of:	LPA	MVPA	ST	Total wear-time
LPA	--	0.17 [-1.49, 1.84]	0.28 [-0.19, 0.75]	0.06 [-0.29, 0.41]
MVPA	-0.01 [-0.09, 0.06]	--	0.003 [-0.06, 0.07]	0.01 [-0.05, 0.08]
ST	-0.28 [-0.75, 0.19]	-0.003 [-0.07, 0.06]	--	**0.02 [0.01, 0.03]** **
		WHR		
	With 30 min/day of:			
Replacing 30 min/day of:	LPA	MVPA	ST	Total wear-time
LPA	--	-0.002 [-0.02, 0.02]	0.004 [-0.003, 0.01]	0.001 [-0.004, 0.01]
MVPA	0.002 [-0.02, 0.02]	--	0.01 [-0.01, 0.02]	-0.001 [-0.02, 0.02]
ST	-0.004 [-0.01, 0.003]	-0.01 [-0.02, 0.01]	**--**	**0.004 [0.001, 0.01]** **
		BF% [b]		
	With 30 min/day of:			
Replacing 30 min/day of:	LPA	MVPA	ST	Total wear-time
LPA	--	-0.16 [-3.87, 3.55]	0.73 [-0.36, 1.81]	-0.007 [-0.80, 0.78]
MVPA	0.16 [-3.55, 3.87]	--	0.89 [-2.22, 3.99]	-0.17 [-3.51, 3.17]
ST	-0.73 [-1.81, 0.36]	-0.89 [-3.99, 2.22]	--	**0.72 [0.15, 1.28]** *

Bold estimates represent significant or marginally significant associations. Abbreviations: min: minutes; CI: confidence interval; LPA: light physical activity; MVPA: moderate-to-vigorous physical activity; ST: sedentary time; BMI: body mass index; WHR: waist-to-height ratio; BF%: body fat percent. All models were adjusted for child age, ethnicity, mother's education, and accelerometer wear-time. [a] Log BMI was used to satisfy model assumptions. Estimates are presented in the log transformation of BMI. [b] n = 71; ** $p < 0.01$; * $p < 0.05$.

Table S1c. Isotemporal substitution associations (β [95% CI]) using 15-minute reallocations between 30-month changes in non-school time LPA, MVPA, and ST and BMI, WHR, and BF% at 30-month follow-up in boys (n = 63).

		BMI [a]		
	With 30 min/day of:			
Replacing 30 min/day of:	LPA	MVPA	ST	Total wear-time
LPA	--	-0.05 [-0.13, 0.03]	-0.02 [-0.05, 0.01]	0.01 [-0.01, 0.04]
MVPA	0.05 [-0.03, 0.13]	--	0.03 [-0.03, 0.09]	-0.04 [-0.10, 0.03]
ST	0.02 [-0.01, 0.05]	-0.03 [-0.09, 0.03]	--	-0.01 [-0.02, 0.004]
		WHR		
	With 30 min/day of:			
Replacing 30 min/day of:	LPA	MVPA	ST	Total wear-time
LPA	--	-0.01 [-0.04, 0.01]	-0.003 [-0.01, 0.01]	0.002 [-0.01, 0.01]
MVPA	0.01 [-0.01, 0.04]	--	0.01 [-0.01, 0.03]	-0.01 [-0.03, 0.01]
ST	0.003 [-0.01, 0.01]	-0.01 [-0.03, 0.01]	--	-0.001 [-0.003, 0.002]
		BF% [b]		
	With 30 mins/day of:			
Replacing 30 min/day of:	LPA	MVPA	ST	Total wear-time
LPA	--	**-3.71 [-7.72, 0.30]** #	-0.82 [-2.19, 0.55]	0.62 [-0.54, 1.78]
MVPA	**3.71 [-0.30, 7.72]** #	--	**2.89 [-0.18, 5.96]** #	**-3.09 [-6.28, 0.09]** #
ST	0.82 [-0.55, 2.19]	**-2.89 [-5.96, 0.18]** #	--	-0.20 [-0.70, 0.30]

Bold estimates represent significant or marginally significant associations. Abbreviations: min: minutes; CI: confidence interval; LPA: light physical activity; MVPA: moderate-to-vigorous physical activity; ST: sedentary time; BMI: body mass index; WHR: waist-to-height ratio; BF%: body fat percent. All models adjusted for child age, ethnicity, mother's education, activity, and accelerometer wear-time at baseline. [a] Log BMI was used to satisfy model assumptions. Estimates are presented in the log transformation of BMI. [b] n = 57 # $p < 0.10$.

Table S1d. Isotemporal substitution associations (β [95% CI]) using 15-minute reallocations between 30-month changes in non-school time LPA, MVPA, and ST and BMI, WHR, and BF% at 30-month follow-up in girls (n = 71).

BMI [a]				
	With 30 min/day of:			
Replacing 30 min/day of:	LPA	MVPA	ST	Total wear-time
LPA	--	0.01 [-0.07, 0.09]	0.01 [-0.01, 0.04]	-0.001 [-0.02, 0.02]
MVPA	-0.01 [-0.09, 0.07]	--	0.001 [-0.07, 0.07]	0.01 [-0.06, 0.09]
ST	-0.01 [-0.04, 0.01]	-0.001 [-0.07, 0.07]	--	**0.01 [0.002, 0.03]** *
WHR				
	With 30 min/day of:			
Replacing 30 min/day of:	LPA	MVPA	ST	Total wear-time
LPA	--	-0.003 [-0.03, 0.02]	0.003 [-0.004, 0.01]	0.001 [-0.01, 0.01]
MVPA	0.003 [-0.02, 0.03]	--	0.01 [-0.02, 0.03]	-0.001 [-0.02, 0.02]
ST	-0.003 [-0.01, 0.004]	-0.01 [-0.03, 0.02]	--	**0.004 [0.001, 0.01]** *
BF% [b]				
	With 30 min/day of:			
Replacing 30 min/day of:	LPA	MVPA	ST	Total wear-time
LPA	--	0.21 [-3.65, 4.07]	0.60 [-0.61, 1.82]	-0.03 [-0.93, 0.87]
MVPA	-0.21 [-4.07, 3.65]	--	0.39 [-2.84, 3.63]	0.18 [-3.29, 3.65]
ST	-0.60 [-1.82, 0.61]	-0.39 [-3.63, 2.84]	--	**0.57 [-0.05, 1.20]** #

Bold estimates represent significant or marginally significant associations. Abbreviations: min: minutes; CI: confidence interval; LPA: light physical activity; MVPA: moderate-to-vigorous physical activity; ST: sedentary time; BMI: body mass index; WHR: waist-to-height ratio; BF%: body fat percent. All models adjusted for child age, ethnicity, mother's education, activity, and accelerometer wear-time at baseline. [a] Log BMI was used to satisfy model assumptions. Estimates are presented in the log transformation of BMI. [b] n = 65; * $p < 0.05$; # $p < 0.10$.
